# Defining the Clinical Phenotypic Ground Truth: Expert Interrater Agreement in Hyperkinetic Movement Disorders

**DOI:** 10.1002/mdc3.70808

**Published:** 2026-10-05

**Authors:** Madelein van der Stouwe, Olof Vermeulen, Wieke Eggink, Tove Henriksen, Eavan McGovern, Marenka Smit, Selma Aybek, Agnita Boon, Rick Brandsma, Andres Deik, Roberto Erro, Alberto Espay, Cristian Falup‐Pecurariu, Stephen Frucht, Christos Ganos, Emilia Gatto, Rick Helmich, Christine Klein, Hans Koelman, Vincent Odekerken, Lisette Koens, Abhimanyu Mahajan, Francesca Morgante, Alexander Münchau, Pattamon Panyakaew, Kathryn Peall, Bélen Pérez‐Dueñas, Emanuel Roze, Anna Sadnicka, Susanne Schneider, Petra Schwingenschuh, Tereza Serranova, Matej Skorvanek, Maria Stamelou, Gertrúd Tamas, Michele Tinazzi, Anna Valadas, Wim Vandenberghe, Jeroen de Vries, Rodi Zutt, Sterre van der Veen, Rogier Luiken, Inge Tuitert, Marina Tijssen

**Affiliations:** ^1^ Expertise Center Movement Disorders Groningen University Medical Center Groningen Groningen the Netherlands; ^2^ Department of Neurology University Medical Center Groningen, University of Groningen Groningen the Netherlands; ^3^ Department of Neurology Ommelander Ziekenhuis Groningen Scheemda the Netherlands; ^4^ Movement Disorder Clinic University Hospital of Bispebjerg Copenhagen Denmark; ^5^ Department of Neurology Beaumont Hospital Dublin Ireland; ^6^ School of Medicine Royal College of Surgeons in Ireland Dublin Ireland; ^7^ Department of Neurology Medical Clinic Velsen Velsen‐Noord the Netherlands; ^8^ Department of Neurology, Faculty of Science and Medicine University of Fribourg Fribourg Switzerland; ^9^ Psychosomatic Medicine Unit, Department of Neurology Inselspital Bern University Hospital, University of Bern Bern Switzerland; ^10^ Department of Neurology Erasmus MC Rotterdam Rotterdam the Netherlands; ^11^ Department of Pediatric Neurology University Medical Center Utrecht Utrecht the Netherlands; ^12^ Parkinson's Disease and Movement Disorders Center University of Pennsylvania Philadelphia Pennsylvania USA; ^13^ Department of Medicine Surgery and Dentistry “Scuola Medica Salernitana,” University of Salerno Fisciano Italy; ^14^ James J. and Joan A. Gardner Family Center For Parkinson's Disease and Movement Disorders University of Cincinnati Cincinnati Ohio USA; ^15^ Faculty of Medicine Transilvania University Brasov Romania; ^16^ Department of Neurology County Clinic Hospital Brasov Romania; ^17^ NYU Grossman School of Medicine New York New York USA; ^18^ Division of Neurology, Department of Medicine, Movement Disorders Clinic Toronto Western Hospital, UHN, University of Toronto Toronto Ontario Canada; ^19^ Krembil Brain Institute Toronto Ontario Canada; ^20^ Department of Clinical Medicine Sanatorio de la Trinidad Mitre, Affiliated to the Faculty of Medicine of the University of Buenos Aires Buenos Aires Argentina; ^21^ Parkinson's and Movement Disorders Section of the Institute of Neurosciences Buenos Aires Buenors Aires Argentina; ^22^ Centre for Cognitive Neuroimaging, Donders Institute for Brain, Cognition and Behaviour, Radboud University Nijmegen Nijmegen the Netherlands; ^23^ Department of Neurology, Center of Expertise for Parkinson and Movement Disorders, Donders Institute for Brain, Cognition and Behaviour Radboud University Medical Centre Nijmegen the Netherlands; ^24^ Institute of Neurogenetics University of Lübeck Lübeck Germany; ^25^ Department of Neurology Amsterdam University Medical Centers, University of Amsterdam Amsterdam the Netherlands; ^26^ Department of Neurology and Clinical Neurophysiology Martini Ziekenhuis Groningen the Netherlands; ^27^ Neurosciences and Cell Biology Institute Neuromodulation and Motor Control Section, City St George's University of London London UK; ^28^ Institute of Systems Motor Science, CBBM, University of Lübeck Lübeck Germany; ^29^ Chulalongkorn Centre of Excellence for Parkinson's Disease and Related Disorders, Division of Neurology, Department of Medicine, Faculty of Medicine King Chulalongkorn Memorial Hospital and Chulalongkorn University, Thai Red Cross Society Bangkok Thailand; ^30^ Laboratories of Neuroscience Research, Division of Psychological Medicine and Clinical Neurosciences Cardiff University Cardiff UK; ^31^ Neuroscience and Mental Health Innovation Institute Cardiff University Cardiff UK; ^32^ Innovative Therapies in Pediatric Neurology Research Group Vall d'Hebron Research Institute Barcelona Spain; ^33^ Department of Pediatrics Universitat Autònoma de Barcelona Barcelona Spain; ^34^ Sorbonne University, INSERM, CNRS, Paris Brain Institute, AP‐HP, Institute of Neurology Paris France; ^35^ Gatsby Computational Neuroscience Unit, University College London London UK; ^36^ Department of Clinical and Movement Neurosciences University College London London UK; ^37^ National Hospital for Neurology and Neurosurgery London UK; ^38^ INSPIRE‐PNRM+, Neuroimaging Center (NIC), University Medical Center of the Johannes Gutenberg University Mainz Mainz Germany; ^39^ Department of Neurology Medical University of Graz Graz Austria; ^40^ Department of Neurology 1st Faculty of Medicine and General University Hospital in Prague, Charles University Prague Czech Republic; ^41^ Department of Neurology P.J. Safarik University Kosice Slovak Republic; ^42^ Department of Neurology University Hospital of L. Pasteur Kosice Slovak Republic; ^43^ Parkinson's Disease and Movement Disorders Department HYGEIA Athens Greece; ^44^ Department of Neurology Semmelweis University Budapest Hungary; ^45^ Biomedicine and Movement Sciences, Neurology Unit, Department of Neurosciences University of Verona Verona Italy; ^46^ Department of Neurosciences and Mental Health Neurology Hospital Santa Maria, CHLUN Lisbon Portugal; ^47^ Faculty of Medicine, Instituto de Medicina Molecular João Lobo Antunes University of Lisbon Lisbon Portugal; ^48^ Department of Neurology University Hospitals Leuven Leuven Belgium; ^49^ Department of Neurosciences KU Leuven Leuven Belgium; ^50^ Department of Neurology HagaZiekenhuis The Hague the Netherlands; ^51^ Department of Rehabilitation Medicine University Medical Center Groningen, University of Groningen Groningen the Netherlands

**Keywords:** tremor, myoclonus, dystonia, functional movement disorders, interrater agreement

## Abstract

**Background:**

Reliable clinical phenotyping is crucial for diagnosis, treatment, and research in hyperkinetic movement disorders, yet its interrater reliability remains uncertain.

**Objectives:**

To quantify interrater agreement in clinical phenotyping of hyperkinetic movement disorders among experts in a large, phenotypically diverse cohort.

**Methods:**

Within the NEMO project, 161 patients with hyperkinetic movement disorders underwent standardized video assessments. Each patient was independently classified by three international movement disorders experts in a structured, stepwise procedure. Experts first assessed videos alone, then received clinical history and examination, and finally diagnostic test results. At each step, they assigned primary and secondary phenotypes and rated their diagnostic confidence. Interrater agreement was assessed using Fleiss’ and Cohen's kappa.

**Results:**

Across all phenotypes, interrater agreement on the primary phenotype increased with added information (*κ* = 0.457 to *κ* = 0.607). Agreement with the NEMO reference classification similarly improved (*κ* = 0.478 to *κ* = 0.656). Among patients with a single dominant phenotype, agreement reached *κ* = 0.78. In 93% of cases, at least two out of three experts reached consensus. Agreement was substantially lower in patients with mixed or complex presentations, such as myoclonus‐dystonia and functional movement disorders. Diagnostic certainty rose throughout, though secondary phenotype agreement remained poor. Experts rarely revised initial impressions, suggesting robust pattern recognition but also potential confirmation bias.

**Conclusions:**

Movement disorders specialists can reliably identify hyperkinetic phenotypes, even without in‐person assessment. While complex, mixed and functional presentations remain diagnostically challenging, our findings reinforce the value of expert phenotyping in clinical care, training, and research, and provide a benchmark for future studies and computational approaches.

Movement disorders such as tremor, myoclonus, dystonia, chorea, and tics are all characterized by excessive involuntary movements. These movements hinder patients in their daily activities and negatively impact their quality of life. To the untrained eye, these hyperkinetic movement disorders may all appear similar. But to the trained eye of a neurologist—and especially to movement disorders experts—careful observation and classification of the phenotype are the cornerstone of patient care. Phenotyping shapes every step of the diagnostic trajectory: it informs decisions to conduct further laboratory testing, clinical neurophysiological examination, brain imaging, or genetic analysis.^1^, ^2^, ^3^ As pharmacological and surgical treatments in movement disorders are typically symptomatic and phenotype‐specific, accurate classification is essential not only to the diagnosis, but to clinical outcomes. Deep brain stimulation, for instance, relies fundamentally on the movement disorder phenotype to select the optimal anatomical target.^4^

As Sadnicka and Edwards have recently pointed out, thousands of disease processes can lead to just a handful of movement disorder phenotypes.^5^ These core phenotypes (tremor, myoclonus, dystonia, tics, chorea, bradykinesia) each seem to represent a stable pattern of disordered movement with distinct clinical features. They propose that this convergence, in which many different conditions give rise to a limited number of phenotypes, suggests that the sensorimotor system may only “break” in a limited number of ways: an insight that underscores the value of phenotype classification in both clinical care and mechanistic research. That said, in certain clinical scenarios, accurate phenotypic classification itself serves as a shortcut to etiological diagnosis: recognizing alcohol‐responsive myoclonus‐dystonia as a distinct syndrome, for instance, immediately directs the clinician toward targeted genetic investigation rather than prolonged debate over the precise phenomenological boundaries between myoclonus and dystonia. In most clinical situations, careful phenotyping remains the essential first step in guiding the diagnostic workup.

Phenotyping also holds a central place in the education of neurologists. Yet it remains a skill that resists easy quantification: shaped not by rule‐following but by pattern recognition developed through experience, feedback and close mentorship with movement disorders specialists. In research, the need for robust and reproducible phenotyping is becoming increasingly urgent. Advances in pathophysiology, genetics, and computational neurology depend on reliable clinical labels.^6^ Whether in imaging studies, biomarker discovery, or training machine learning models, the quality of any analysis is fundamentally constrained by the accuracy of the phenotypic input.

Despite its importance, the reliability of expert‐based phenotyping is not well quantified. What level of agreement can be expected among movement disorder experts? Most clinicians will have experienced a spirited debate between two specialists over whether a case is best described as “tremor” or “myoclonus”—a reminder that even expert opinion is subject to variation.

Several prior studies have investigated interrater agreement on movement disorder phenotyping, yet often in limited populations. Three studies focused on functional movement disorders. Van der Salm et al. assessed agreement among experts evaluating 60 patients with myoclonus, tics, and functional jerks, reporting moderate agreement (*κ* = 0.56; *κ* ranges from −1 to 1, with values near 1 indicating strong agreement and values near 0 reflecting chance‐level agreement^7^).^8^ Morgante et al found only fair agreement among both movement disorder and general neurologists classifying 28 patients with either functional or non‐functional movement disorders, with improvement after access to clinical information.^9^ In the context of functional versus primary tics, Rigas et al. reported fair agreement based on video assessment of 24 patients (*κ* = 0.21), which improved to moderate (*κ* = 0.51) with additional clinical context.^10^ Two other studies have examined pediatric populations. Eggink et al. found modest agreement (*κ* = 0.35) in children with spasticity, dystonia and ataxia,^11^ while Yilmaz et al demonstrated significant discordance among pediatric neurologists, particularly when multiple phenotypes coexisted.^12^

Other studies have illustrated the diagnostic gray zones movement disorders specialists regularly encounter: Becktepe et al and Du et al both highlighted the particular difficulty of dystonia‐adjacent cases: agreement on tremor syndromes varied by subspecialty, and subtle dystonic features in essential tremor patients were prone to divergent interpretation.^13^, ^14^ Fearon et al further highlighted these challenges in the assessment of soft signs, showing that even among movement disorder specialists, agreement on the presence of subtle neurological abnormalities was only fair to moderate (*κ* = 0.31–042).^15^

If phenotyping is to support therapeutic development and mechanistic research its reliability must be demonstrable. Therefore, the current study builds on earlier work by examining interrater agreement in a large and diverse cohort of hyperkinetic movement disorders. Leveraging the data infrastructure of the Next Move in Movement Disorders (NEMO) project,^16^ we use a stepwise expert assessment design that includes a structured video protocol, clinical history, and diagnostic test results.^17^ This approach allows us to evaluate phenotyping reliability across both clearly recognizable and diagnostically challenging cases, with the aim of setting a benchmark for phenotyping reliability and to support efforts toward standardization in both clinical and research contexts.

## Methods

This study was conducted at the Expertise Centre for Movement Disorders at the University Medical Center Groningen (UMCG), within the NEMO project.^17^, ^18^ The broader aim is to develop a machine learning–assisted tool to support clinical phenotyping in movement disorders. The present substudy focuses on the reliability of expert‐based visual classification using standardized clinical video assessments combined with additional clinical information. The study was approved by the Medical Ethical Committee of the UMCG (METc 2018‐444) and conducted in accordance with the Declaration of Helsinki.

### Patients

Patients were eligible for inclusion if they were aged ≥16, and had a clinically confirmed hyperkinetic movement disorder with involvement of the upper limbs. Recruitment was carried out through the expertise centers outpatient clinic and patient database, and was extended to affiliated hospitals and patient associations where necessary. All patients provided written informed consent prior to enrolment.

Recruitment was structured around two broad phenomenological tiers. The first comprised patients with a single dominant movement disorder phenotype: tremor, myoclonus, dystonia, ataxia, chorea, tics, spasticity or functional movement disorders—mirroring the classification options present to expert raters. Within this tier, specific clinical entities were selected as representative examples: essential tremor, enhanced physiological tremor, cortical myoclonus, benign idiopathic myoclonus, isolated dystonia and jerky/tremulous functional movement disorders. The second tier comprised patients with combined or mixed phenotype, for whom myoclonus‐dystonia (with and without a confirmed *SGCE* mutation) served as the primary example. We aimed to include >20 patients in the groups of tremor, myoclonus and dystonia in the first tier, with a smaller number of patients with chorea, tics, ataxia, spasticity, dystonic tremor, parkinsonian tremor, and enhanced physiological tremor to reflect the diversity encountered in clinical practice. The group sizes were determined in part by the requirements of the broader NEMO project, which aimed to generate sufficient data for machine learning model development. Spasticity was included given the clinical overlap with dystonia that can make differentiation challenging; dystonic tremor and parkinsonian tremor were included to reflect the full spectrum of tremor presentations encountered in a tertiary movement disorders clinic, rather than restricting the cohort to phenotypically unambiguous cases.

### Assessment Protocol

All patients underwent the full, standardized NEMO assessment protocol including clinical data collection, and video recordings.^17^ Video data were acquired using the Intel® RealSense™ D435 depth‐sensing camera, which captures synchronized RGB and depth video (3D).

Each patient was recorded while performing a fixed sequence of 24 movement tasks selected to evoke a broad range of hyperkinetic movement disorders.^19^ These tasks included rest, maintenance of various postures, goal‐directed actions, repetitive movements such as finger tapping, and performance under cognitive distraction. All recordings were conducted by trained instructors following a standardized protocol to ensure consistency.

Clinical and demographic data were collected using REDCap electronic data capture tools hosted at the UMCG.^20^ Collected variables included age, sex, age at symptom onset, symptom duration, medication use, affected body regions, contributing factors and comorbidities. Additional data relevant to the diagnostic work‐up—including findings from clinical neurophysiology, neuroimaging, and genetic testing—were extracted from the patients' clinical records and also entered into REDCap. These data were later on used to supplement the video‐based ratings in a stepwise fashion.

### Expert Ratings

Expert assessment was carried out by an international panel of movement disorders specialists (authors HE‐RZ). Experts were invited to participate based on predefined criteria: all are experienced movement disorders specialists with recognized clinical and academic expertise in hyperkinetic movement disorders, and membership of the MDS. The final panel was selected following discussion between the senior investigator (AMMS and MdK), with the aim of ensuring international representation and broad subspecialty coverage. Experts were asked to assess between 10 and 15 patients; some participated twice as the study progressed. Every patient was independently rated by three different experts. To avoid bias, experts were blinded to the a priori classification assigned by the NEMO research team, which was made based on all available information.

Assessments were conducted in a structured, stepwise fashion (Fig. 1). Step 1 involved rating based on video recordings alone. In step 2, clinical information was added, simulating what a neurologist would typically have after a first consultation. This included a brief medical history, clinical information such as age and mode of onset, findings from general neurological examination, and a sample of handwriting and spiral drawing. In the final step 3, additional diagnostic data were provided, including results from clinical neurophysiology, neuroimaging, and genetic testing if available. At each of these three steps, experts were asked to determine (a) the primary movement disorder phenotype (choices: tremor, myoclonus, dystonia, ataxia, chorea, tics, spasticity, functional movement disorder); (b) whether a secondary phenotype was present (same options plus “none”); and (c) how confident they were in their phenotyping decision on a scale from 0% to 100%.

**Figure 1:**
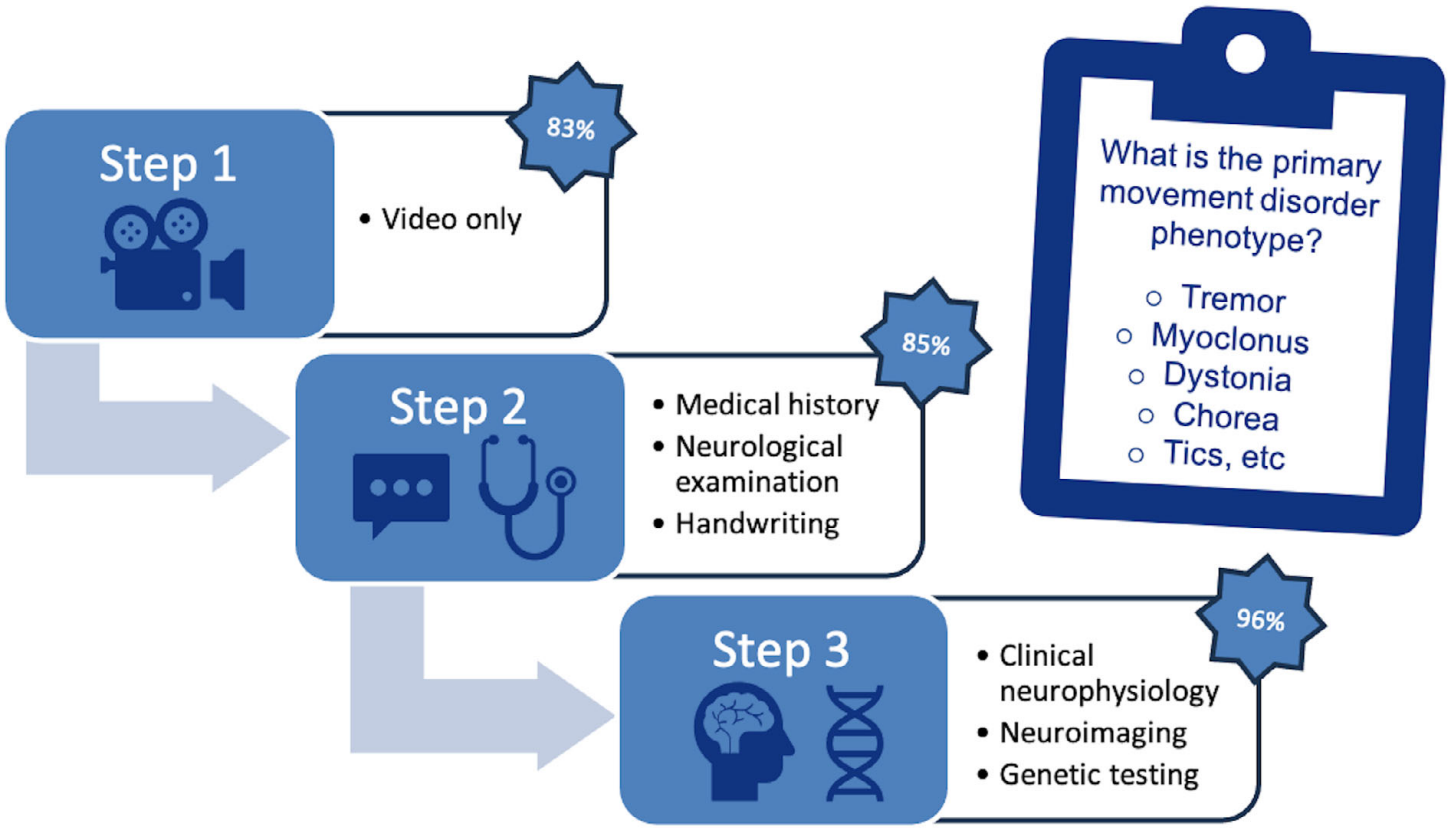
Flowchart depicting the stepwise expert assessment procedure. Diagnostic certainty (%) is shown in the blue stars for each step.

### Statistics

All statistical analyses were performed using IBM SPSS Statistics, version 28 (IBM Corp., Armonk, NY, USA). To evaluate interrater agreement on the primary movement disorder phenotype among experts, Fleiss’ kappa was calculated for each of the three diagnostic steps. Interpretation of kappa values followed standard thresholds: values *κ* < 0.20 were considered slight agreement, 0.21–0.40 fair, 0.41–0.60 moderate, 0.61–0.80 substantial, and >0.80 almost perfect agreement.^21^ We analyzed the interrater agreement on the primary movement disorder phenotype among experts across the full cohort. In addition, observed agreement on the primary phenotype was also reported as the percentage of cases in which 2/3 or all three experts assigned the same phenotype. Agreement between individual expert classifications and the reference phenotype assigned by the NEMO team was assessed using Cohen's kappa for each step, using the same thresholds for interpretation as for Fleiss’ kappa. Diagnostic certainty was summarized per step and tested for change over the course of the rating process using Friedman's analysis of variance, and, in case of significant differences, subsequent pairwise comparisons using Bonferroni corrected significance values. Similarly, we investigated whether diagnostic certainty differed in cases dependent on the observed agreement among experts using an independent‐samples Kruskal‐Wallis test with pairwise comparisons. Agreement on the secondary movement disorder phenotype was evaluated using Fleiss’ *κ* at each diagnostic step. Finally, we recorded the number of diagnostic changes made by each expert between steps to further explore the effect of added clinical information on classification stability. *P* < 0.05 were considered statistically significant. It should be noted that kappa values are sensitive to the prevalence distribution of categories in the sample; in unbalanced cohorts, kappa may underestimate observed agreement. Observed agreement percentages are therefore reported alongside kappa values to also provide a more intuitive measure. The reference phenotype assigned by the NEMO team represents a clinical consensus classification based on all available information, rather than an independent biological gold standard. As no such gold standard exists for the majority of hyperkinetic movement disorders, Cohen's kappa in this context reflects agreement with the best available clinical reference rather than diagnostic accuracy in an absolute sense.

As a post hoc analysis, we also examined interrater agreement on the primary movement disorder phenotype among experts in three clinically relevant subgroups: patients with single phenotypes only, patients with myoclonus dystonia (as an example of a mixed phenotype), and patients with functional jerky or tremulous movement disorders (as a potentially diagnostically challenging group). These subgroup classifications were assigned by the NEMO research team based on clinical consensus among the team prior to expert assessment. The post hoc analyses were performed to explore whether agreement patterns differed in phenotypically more or less complex cases, and to support interpretation of the findings across distinct clinical scenarios.

## Results

A total of 161 patients were included in this study, covering a broad range of hyperkinetic movement disorders. Patients included those exhibiting tremor (n = 49; clinical diagnoses included essential tremor (n = 29), enhanced physiological tremor (n = 10), parkinsonian tremor (n = 8), Holmes tremor (n = 2)), myoclonus (n = 26: cortical myoclonus (n = 22), benign idiopathic myoclonus (n = 4)), dystonia (n = 22), tics (n = 2), chorea (n = 2), ataxia (n = 2), and spasticity (n = 2). Among the patients with myoclonus‐dystonia (n = 30), 17 had a confirmed *SGCE*‐mutation and 13 did not. Four patients were included with dystonic tremor. Finally, a group of patients with jerky/tremulous functional movement disorders (n = 22) was included. Demographic and clinical characteristics of the included patients are presented in Table 1. A total of 38 international movement disorders experts participated, each assessing a median of 11 patients (IQR 10–13, range 7–30), resulting in 483 assessments.

**TABLE 1:** Clinical characteristics and test results

		Tremor (n = 49)	Myoclonus (n = 26)	Dystonia (n = 22)	Myoclonus dystonia (n = 30)	Tics (n = 2)	Chorea (n = 2)	Ataxia (n = 2)	Spasticity (n = 2)	FMD (n = 22)
Age		65 (25) range 19–81	24.5 (20) range 16–77	46 (36) range 16–76	27 (40) range 17–75	31 range 31–31	55.5 range 37–74	63.5 range 62–65	46 range 30–62	63 (23) range 25–75
Female sex		31%	69%	50%	34%	50%	50%	50%	50%	41%
Age at onset		39 (44)	12 (15)	14 (35)	6 (8)	5	30	52	21.5	45.5 (29)
Disease duration		13 (30.5)	13 (9.5)	16 (16)	23 (36.5)	26	25.5	11.5	24.5	12.5 (21.5)
Family history of movement disorders		59%	31%	18%%	83%	100%	50%	100%	0%	52%
Polymyography	Fits with phenotype	17	20	10	8	0	0	0	0	13
	Not performed	32	6	12	22	2	2	2	2	11
EEG–EMG	Cortical origin	0	12	0	2	0	0	0	0	0
	No cortical origin	0	8	0	5	0	0	0	0	1
	Not performed	49	6	22	23	2	2	2	2	21
Brain MRI	Relevant lesions	2	2	1	0	0	0	1	2	0
	No relevant lesions	12	12	12	3	0	1	0	0	6
	Not performed	35	12	9	27	2	1	1	0	16
FDOPA‐PET	Dopaminergic deficit	6	0	0	0	0	0	0	0	0
	Normal	5	0	1	0	0	0	0	0	0
	Not performed	38	26	21	30	2	2	2	2	22
Genetic testing	Relevant variant	1	8	4	18	0	1	2	0	0
	No relevant variants	4	11	7	6	0	1	0	0	0
	Not performed	44	7	11	6	2	0	0	2	22
	Genes	PARK‐*GBA1*	ATX/MYC‐*NUS1*, n = 2 ATX‐*MT‐ATP6* MARCH6 SCN1A, n = 2 STARD7 16p11.2	DYT‐*TOR1A* DYT‐*KMT2B* DYT‐*THAP1* DYT‐*TUBB4A*	MYC/DYT‐*SGCE*, n = 17 Silver Russel syndrome	‐	CHOR‐*NKX2‐1*	ATX‐*ATXN3*, n = 2	‐	‐

Clinical characteristics are reported as median (interquartile range) or percentage, test results as counted frequencies. FMD: functional movement disorder. Genes are listed in line with the International Parkinson and Movement Disorders Society genetic nomenclature where possible.

### Interrater Agreement among Experts

Expert interrater agreement on the primary phenotype ranged from a Fleiss’ *κ* of 0.457 based on videos only, to 0.525 after receiving additional clinical information, and 0.607 after disclosing all diagnostic test results (Fig. 2).

**Figure 2:**
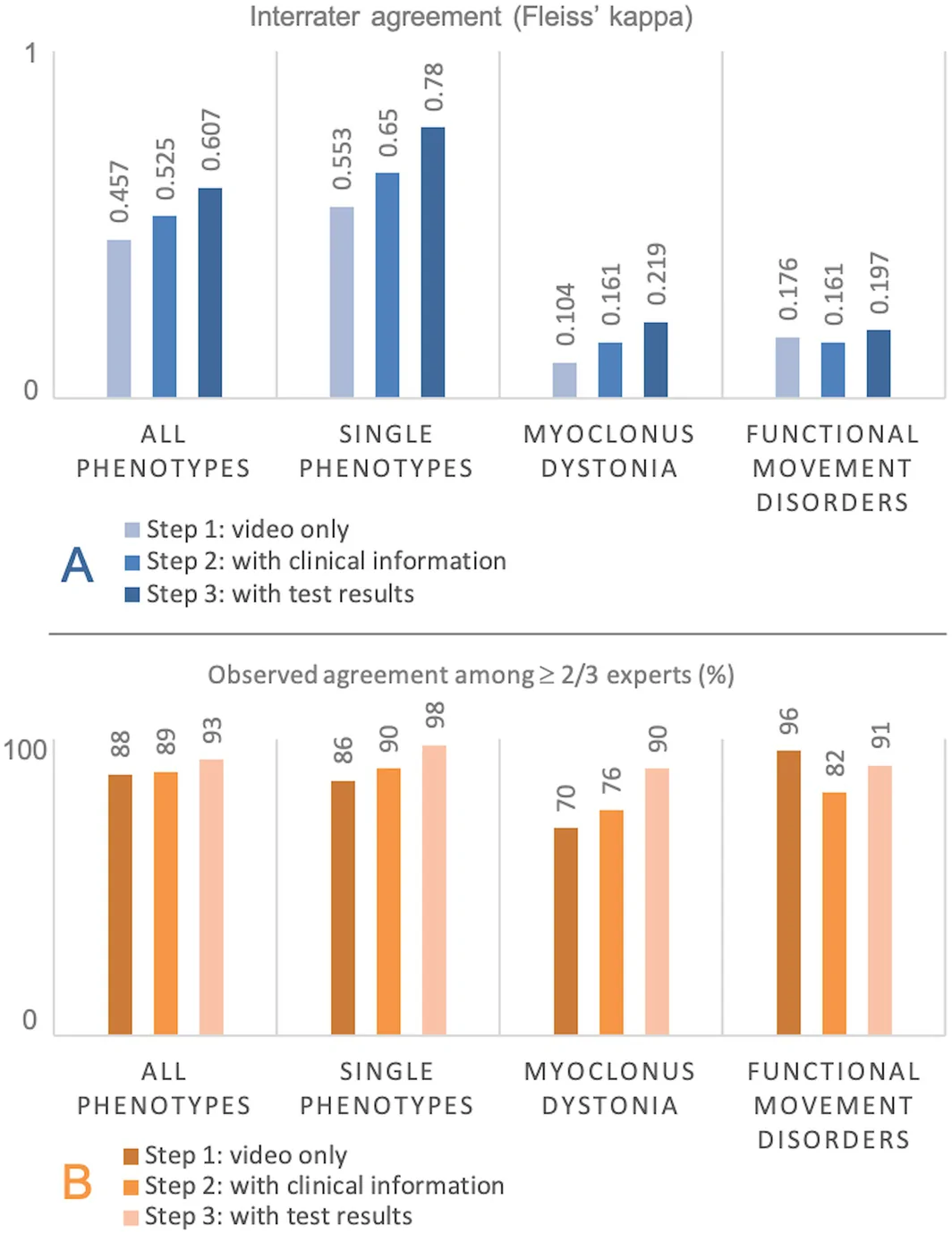
(A) Interrater agreement on the primary phenotype shown as Fleiss’ kappa and (B) observed agreement among ≥2/3 experts on the primary phenotype shown in percentages, for each diagnostic step, for the overall analysis including all phenotypes and for three post hoc analyses separately.

### Observed Agreement among Experts

Full agreement on the primary phenotype among 3/3 experts ranged from 24% of patients based on videos only, 25% after receiving additional clinical information, to 27% upon receiving all diagnostic test results. Partial agreement among 2/3 experts ranged from 88% based on videos only, 89% with additional clinical information, and 93% after receiving all diagnostic test results (Fig. 2).

### Interrater Agreement between Experts and the NEMO Team

Assessment of the interrater agreement between expert classifications and the reference primary phenotype assigned by the NEMO team resulted in a Cohen's *κ* of 0.478 based on videos only, 0.566 after additional clinical information, and 0.656 after receiving all diagnostic test results.

### Diagnostic Certainty

When asked to rate the perceived certainty of their primary phenotype classification, the experts’ certainty increased from a median 83% (Q1: 65%–Q3: 95%) in step 1, to 85% (70%–96%, *p* < 0.001) in step 2, to 92% (77%–100%, *p* < 0.001) in step 3 (Fig. 3). Diagnostic certainty increased with expert agreement: 78% in cases with no agreement, 87% in cases with 2/3 agreement and 96% in cases with 3/3 agreement (*p* < 0.001).

**Figure 3:**
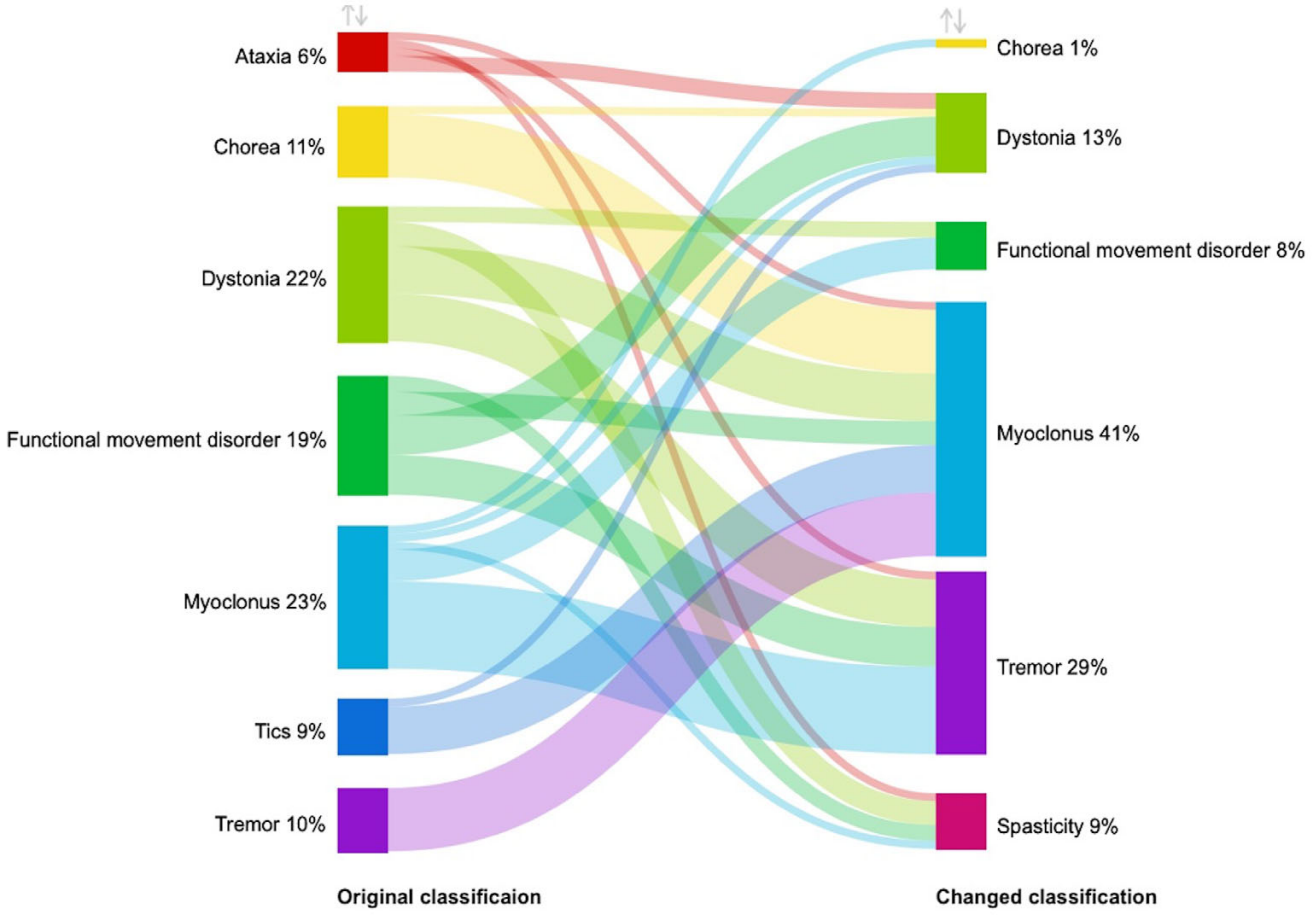
Sankey plot depicting changes in classification (n = 113) of the primary movement disorder phenotype across the entire assessment process, with the experts' original classification on the left and the changed classification on the right.

### Secondary Phenotypes

Experts identified a secondary phenotype in 39% of patients in step 1, 41% in step 2, and 42% in step 3. Interrater agreement among experts on the secondary phenotype was slight, as indicated by *κ* = 0.085 in step 1, 0.101 in step 2, and 0.091 in step 3.

### Changes in Classification

Experts could change their phenotype classification twice: 12% of classifications were changed after receiving additional clinical information, whereas 11% of classifications were changed upon receiving all diagnostic test results. In total, this resulted in a total of 113 changes in classification, with most changes in primary phenotype between tremor and myoclonus (23%), from chorea, dystonia and tics to myoclonus (25%), and from functional movement disorders to dystonia, tremor, myoclonus and spasticity (19%) (Fig. 3).

### Post Hoc Subgroup Analyses

In the subgroup of patients with a single dominant movement disorder phenotype (n = 95), interrater agreement on the primary phenotype increased across steps from *κ* = 0.553, to 0.650, to 0.780. This subgroup comprised patients with a dominant phenotype of tremor (essential tremor [n = 29], enhanced physiological tremor [n = 10]), myoclonus (cortical myoclonus [n = 22], benign idiopathic myoclonus [n = 4]), dystonia (isolated dystonia [n = 22], tics [n = 2], chorea [n = 2], ataxia [n = 2], spasticity [n = 2]). Each clinical diagnosis was thus grouped under the corresponding phenomenological category used in the expert classification framework; experts were asked to classify at this phenomenological level, and distinguishing between specific entities within a category, (eg, essential tremor vs. enhanced physiological tremor), was not part of the rating task. For patients with myoclonus dystonia, interrater agreement was lower, ranging from *κ* = 0.104, to 0.161–0.219. In this group, after step 3, 63% of experts selected myoclonus as the primary phenotype, of whom 46% reported no secondary movement disorder and 37% identified dystonia as secondary. Conversely, 18% selected dystonia as the primary phenotype in step 3, of whom 44% chose myoclonus as secondary and 38% no secondary phenotype (see Supplementary Materials for an overview of myoclonus dystonia classifications). In the functional jerky/tremulous movement disorder group, interrater agreement remained low across all steps (*κ* = 0.176, 0.161, and 0.197). After full information disclosure in step 3, 55% of experts identified a functional movement disorder as the primary phenotype, while others chose tremor (21%), myoclonus (8%), dystonia (8%), chorea (6%), or ataxia (3%). Functional movement disorder was selected as a secondary phenotype in only 3% of assessments.

Observed agreement followed a similar pattern. For single phenotypes, full agreement (3/3 experts) increased from 25% to 30% to 32%, whereas partial agreement (at least 2/3 experts) ranged from 86% to 90% to 89%. In the myoclonus dystonia group, full agreement was reached in 7%, 3% and 3% of patients across steps, while partial agreement improved from 70% to 76% to 90%. For functional movement disorders, full agreement remained stable at 18% across all steps, while partial agreement was 96% in step 1, 82% in step 2, and 91% in step 3.

Diagnostic certainty mirrored these trends. In single phenotype patients, median certainty increased from 82% (IQR 69–95%) in step 1, to 87% (70–98%) in step 2 (*p* < 0.001), to 94% (80–100%) in step 3 (*p* < 0.001). For myoclonus dystonia, median certainty was 80% (65–93%) in step 1, 80% (70–95%) in step 2 (*p* = 0.656), and increased to 95% (75–100%) in step 3 (*p* = 0.008). In functional movement disorders, diagnostic certainty remained stable across steps at 80% (61–90%), 81% (64–90%), and 81% (62–94%) (*p* = 0.259).

## Discussion

This study provides a detailed evaluation of interrater agreement on movement disorder phenotyping in the largest and most clinically diverse cohort of patients with hyperkinetic movement disorders to date. Across this spectrum, agreement among movement disorders specialists on the main phenotype after receiving all clinical information was substantial. In 93% of cases, at least two out of three experts independently reached consensus, underscoring the reliability of expert judgment even in the context of video‐based assessments.

These findings extend prior work on interrater agreement on myoclonus, tics and functional movement disorders,^8^, ^9^, ^10^ and on classification of movement disorders in children.^11^, ^12^ Previous studies highlighted the challenges of clinical classification, with kappa values never extending beyond 0.56. In comparison, our data suggest that higher agreement may be more readily observed in broader clinical populations: our cohort included a wide range of hyperkinetic movement disorders, beyond classifications of functional versus non‐functional or complex pediatric presentations, both of which may pose particular diagnostic challenges. With *κ* = 0.61 across the cohort, and *κ* = 0.78 in patients with a single movement disorder phenotype, agreement was substantial, and partial consensus in a least 2/3 experts can nearly always be expected. These findings suggest that our shared language, training, and clinical frameworks support reliable phenotyping of core movement disorder phenotypes. However, this encouraging picture is tempered by the low rate of full agreement across the cohort as a whole, and by the notably poor agreement in mixed and functional presentations, where only slight agreement was reached.

Important differences were found between agreement levels in patients with single phenotypes and those with mixed features. As expected, agreement was highest in cases with a clear single dominant phenotype, such as essential tremor, cortical myoclonus or isolated dystonia, reflected in a Fleiss’ kappa of 0.78, and lower in cases with mixed presentations such as myoclonus‐dystonia and functional movement disorders where only slight agreement was reached (*κ* = 0.22 and 0.20, respectively). These differences highlight the added complexity and subjectivity involved in classifying patients with overlapping symptoms, where even experienced specialists may disagree on their interpretation. This complexity was further reflected in the poor interrater agreement on the secondary movement disorder phenotype, suggesting that experts differed in how they weighed additional features. Together, these results may in part reflect the cognitive limits of visual diagnostic reasoning: experts must integrate multiple simultaneously occurring movement features, of varying prominence and clarity, while drawing on pattern recognition gained from experience. Studies in medical decision‐making suggest that while clinicians are highly skilled at recognizing prototypical presentations, ambiguity or competing cues can reduce consistency.^22^, ^23^, ^24^ In the context of mixed movement disorders, these factors could contribute to differences in interpretation.

The particularly low agreement in functional movement disorders warrants separate consideration. Functional movement disorders occupy an ambiguous position in phenotypic classification: while it is in many respects an etiological diagnosis, it is also associated with specific clinical signs (such as distractibility, variability, and entrainment) that can be considered phenotypic markers in their own right. Recognition of these signs, however, depends on targeted video tasks; while our protocol included relevant tasks such as contralateral tapping and cognitive distraction, we cannot ascertain whether all experts systematically reviewed these specific clips, and furthermore, a standardized fixed‐duration protocol cannot replicate the intuition‐guided flexibility of a clinical examination, where an experienced clinician can extend or adapt distraction tasks until a clear picture emerges. This may have contributed to the low agreement in this group, and is consistent with lower agreement on functional movement disorders reported in prior literature.^8^, ^9^, ^10^ The very low rate of functional movement disorder as a secondary phenotype (identified in only 3% of assessments) further reflects this: in this study, experts were unlikely to identify functional features as an accompaniment to another primary phenotype.

Experts reported high clinical certainty across the assessment process, often already after video‐only evaluation. Certainty increased mildly across the stepwise procedure: from 82% to 93% certainty, except for functional movement disorders where certainty remained at 81% throughout. The combination of stable but modest certainty and persistently low interrater agreement in the functional movement disorders group suggests that individual experts were confident in their own classifications while reaching different conclusions: a pattern consistent with high subjective confidence in the face of genuine diagnostic ambiguity, and one that merits attention in future classification frameworks. Similarly, most experts did not revise their initial classification when clinical information was added: 88–89% of all phenotype classifications remained unchanged when experts were presented with the opportunity to change their mind. This is in line with previous findings by van der Salm et al,^25^ who similarly observed that movement disorders specialists relied predominantly on initial clinical impressions and neurological examination to guide decision‐making. This consistency likely reflects the robustness of pattern recognition among experienced clinicians. At the same time, it raises the possibility that early diagnostic impressions may shape subsequent interpretation, leading to confirmation bias, with clinicians primarily noticing features that confirm their initial classification, at the expense of critical re‐evaluation.^26^ Observer‐level cognitive biases, including anchoring on an initial impression and framing effects where the context in which information is presented influences its interpretation, may contribute to the variability seen across experts, independent of phenotypic complexity. These biases are well recognized in clinical neurology and are relevant to any diagnostic process that relies on sequential information gathering.^27^ Regarding changes in classification, myoclonus emerged as the most reclassified disorder, which highlights how difficult it can be to distinguish myoclonus from other movement disorders, particularly tremor, and emphasizes the importance of neurophysiology in these patients.^28^

It is important to acknowledge that video‐based assessment differs in key ways from in‐person evaluation. Several experts commented that they felt constrained by the inability to interact with patients, repeat tasks, or hear the patient's history in their own words. In‐person assessments also allow for undirected observation, which may reveal signs not captured on video. Additionally, our stepwise information flow (video first, then clinical data) differs from the usual clinical reasoning process. While this format was chosen to foreground phenomenology, even higher agreement might be possible in more naturalistic settings. Still, the substantial agreement between experts and the a priori NEMO classification is encouraging. It suggests that structured video recordings, combined with stepwise clinical information, can provide a sufficiently rich dataset to support reliable phenotyping, reinforcing the validity of video‐based assessments in research settings.

Our study has other limitations. Although the cohort is large and phenotypically diverse, it is not evenly distributed across phenotypes. Disorders such as tremor, myoclonus, dystonia, myoclonus‐dystonia, and functional movement disorders are well represented, while tics, chorea, ataxia, and bradykinesia are underrepresented. This imbalance reflects both the focus areas of our expertise center and the group size requirements of the broader NEMO project, in which adequate representation of the primary phenotype groups tremor, myoclonus and dystonia was prioritized to support machine learning model development. In addition, technical issues affected data quality in some cases. For example, a microphone failure led to missing speech data for part of the cohort, and the use of a 3D camera optimized for depth recording came at the cost of lower‐quality RGB video. Moreover, the quality of video streaming within the VRW depended on users’ internet connection, which may have impacted the rating experience for some experts.

Nonetheless, our study has several strengths as well. It is based on a large and well‐characterized cohort, with structured, extended video and clinical information provided to experts in a controlled, stepwise manner. The use of secure data platforms such as REDCap and the VRW ensured high standards of data integrity and protection. Importantly, our expert panel consisted of experienced raters from different national backgrounds, strengthening the generalizability of our results.

The results provide a reassuring foundation for both clinical practice, training and research. In line with Sadnicka and Edwards,^5^ who emphasize that recognizable “substates” of disordered movement arise across diverse etiologies, our field relies on clinicians learning to identify these stable patterns. Our results support this principle: pattern recognition in movement disorders phenotyping appears to be a teachable and reproducible skill. In cases where diagnostic uncertainty remains, video case conferences—already quite common in clinical practice—may complement individual judgment to strengthen phenotyping reliability. In research contexts, our data support the use of expert consensus as a valid method for patient inclusion. In the NEMO study, we defined reliable phenotypes based on agreement between at least two of three experts and a Fleiss’ *κ* >0.8 across a group.^17^ The results presented here lend empirical support to that approach and may guide similar efforts in future machine learning research.

## Conclusion

This study shows that movement disorders specialists can reliably identify hyperkinetic phenotypes, even in the absence of in‐person assessment. While complex, mixed and functional presentations remain diagnostically challenging, our findings reinforce the value of expert phenotyping in clinical, training and research settings, and provide a benchmark for future studies and computational approaches.

## Author Roles

(1) Research Project: A. Conception, B. Organization, C. Execution; (2) Statistical Analysis: A. Design, B. Execution, C. Review and Critique; (3) Manuscript Preparation: A. Writing of the First Draft, B. Review and Critique.

MS: 1A, 1B, 2A, 2B, 3A.

OV: 1B, 3B.

WE: 1C, 3B.

TH: 1C, 3B.

EMG: 1C, 3B.

M.Smit: 1C, 3B.

SA: 1C, 3B.

AB: 1C, 3B.

RB: 1C, 3B.

AD: 1C, 3B.

RE: 1C, 3B.

AE: 1C, 3B.

CFP: 1C, 3B.

SJF: 1C, 3B.

CG: 1C, 3B.

EG: 1C, 3B.

RH: 1C, 3B.

CK: 1C, 3B.

HK: 1C, 3B.

VO: 1C, 3B.

LK: 1C, 3B.

A.Mahajan: 1C, 3B.

FM: 1C, 3B.

A.Munchau: 1C, 3B.

PP: 1C, 3B.

KP: 1C, 3B.

BP: 1C, 3B.

ER: 1C, 3B.

AS: 1C, 3B.

SS: 1C, 3B.

PS: 1C, 3B.

TS: 1C, 3B.

M.Skorvanek: 1C, 3B.

M.Stamelou: 1C, 3B.

GT: 1C, 3B.

MT: 1C, 3B.

AV: 1C, 3B.

WV: 1C, 3B.

JV: 1C, 3B.

RZ: 1C, 3B.

SV: 1B, 3B.

RL: 1B, 3B.

IT: 1A, 1B, 2C, 3B.

M.Tijssen: 1A, 2A, 2C, 3B.

## Disclosures

**Ethical Compliance Statement:** The study was approved by the Medical Ethical Committee of the UMCG (METc 2018‐444) and conducted in accordance with the Declaration of Helsinki. All patients provided written informed consent prior to enrolment. We confirm that we have read the Journal's position on issues involved in ethical publication and affirm that this work is consistent with those guidelines.

**Financial Disclosures and Conflicts of Interest:** This work was supported by the European Fund for Regional Development of the European Union in collaboration with the province of Fryslan (grant number 01492947), and a ZonMW TOP Grant (grant number 91218013). The authors declare no competing interests relevant to the submitted manuscript.

AMMS reports grant support from ZonMw‐Off Road and ZonMw‐Klinische fellowship. AE has received grant support from the NIH and the Michael J. Fox Foundation; personal compensation as a consultant/scientific advisory board member for Mitsubishi Tanabe Pharma America (formerly, Neuroderm), Amneal, Acorda, Abbvie, Bial, Supernus (formerly, USWorldMeds), NeuroDiagnostics, Inc. (SYNAPS Dx), Intrance Medical Systems, Inc., Merz, Praxis Precision Medicines, Citrus Health, and Herantis Pharma; Data Safety Monitoring Board (chair) of AskBio; and publishing royalties from Lippincott Williams & Wilkins, Cambridge University Press, and Springer. He is co‐inventor of the patent “Compositions and methods for treatment and/or prophylaxis of proteinopathies.” He cofounded REGAIN Therapeutics to fund preclinical studies but relinquished the right to any personal income from future treatments. He serves on the editorial boards of the Journal of Parkinson's Disease, Journal of Alzheimer's Disease, European Journal of Neurology, Movement Disorders Clinical Practice, and JAMA Neurology. FM received research support from NIHR, Innovate UK. Speaking honoraria from Abbvie, Medtronic, Boston Scientific, Bial, Merz; Travel grants from the International Parkinson's disease and Movement Disorder Society; Advisory board fees from Merz and Boston Scientific; Consultancies fees from Boston Scientific, Merz and Bial; Research support from Boston Scientific, Merz; Royalties for the book “Disorders of Movement” from Springer; member of the editorial board of Movement Disorders, Movement Disorders Clinical Practice, European Journal of Neurology, European Journal of Neurology. AM has received funding from the Parkinson's foundation, Sunflower Parkinson disease Foundation and the Dystonia Medical Research Foundation, outside of the submitted work. He served on the scientific advisory board of adaptive biosciences, through the Parkinson's Study Group. He served as a consultant for Vima therapeutics Inc. He receives a stipend for serving as an associate editor for the journal, *Movement Disorders Clinical Practice*. ER received honorarium for speech from Orkyn, Aguettant, Elivie, Merz‐Pharma, Janssen, Teva and for participating in advisory boards from Merz‐Pharma, Elivie, Teva, and BIAL, and he received research support from Merz‐Pharma, Orkyn, Elivie, Everpharma, Aguettant. GT was partly supported by grant No: TKP2021‐EGA‐25. MAJT reports grants from the European Fund for Regional Development from the European Union (01492947) and the province of Friesland, ZonMw‐TOP (91218013), from Stichting Wetenschapsfonds, and unrestricted grants from Merz.

Author disclosures are available in the Supporting Information.

## Supporting information


**Data S1.** Supporting Information.

**Data S2.** Table on myoclonus dystonia classifications.
Each row represents the classifications given by an individual rater: 3 raters for each patient. Myoclonus dystonia, n = 30 patients, 90 assessments.

## Data Availability

The data that support the findings of this study are available from the corresponding author upon reasonable request.
