# Supplementary material for: Defining the Clinical Phenotypic Ground Truth: Expert Interrater Agreement in Hyperkinetic Movement Disorders
Source: Mov Disord Clin Pract. 2026 Oct 5:10.1002/mdc3.70808. Online ahead of print. doi: 10.1002/mdc3.70808 (PMC13636365; doi:10.1002/mdc3.70808)
Supplement: Supplementary file 1 [file MDC3-9999-0-s001.zip › COI_all_authors_checked/Coi Alberto Espay.docx]

| ICMJE DISCLOSURE FORM | |
| --- | --- |
| **Date:** | 1/27/2026 |
| **Your Name:** | Alberto J. Espay |
| **Manuscript Title:** | Defining the Clinical Phenotypic Ground Truth: Expert Interrater Agreement in Hyperkinetic Movement Disorders |
| **Manuscript Number (if known):** | MDCP-26-0004 |
| In the interest of transparency, we ask you to disclose all relationships/activities/interests listed below that are related to the content of your manuscript. “Related” means any relation with for-profit or not-for-profit third parties whose interests may be affected by the content of the manuscript. Disclosure represents a commitment to transparency and does not necessarily indicate a bias. If you are in doubt about whether to list a relationship/activity/interest, it is preferable that you do so.  The author’s relationships/activities/interests should be defined broadly. For example, if your manuscript pertains to the epidemiology of hypertension, you should declare all relationships with manufacturers of antihypertensive medication, even if that medication is not mentioned in the manuscript.  In item #1 below, report all support for the work reported in this manuscript without time limit. For all other items, the time frame for disclosure is the past 36 months. | |

|  | | | **Name all entities with whom you have this relationship or indicate none (add rows as needed)** | **Specifications/Comments (e.g., if payments were made to you or to your institution)** |
| --- | --- | --- | --- | --- |
| **Time frame: Since the initial planning of the work** | | | | |
| **1** | All support for the present manuscript (e.g., funding, provision of study materials, medical writing, article processing charges, etc.)  **No time limit for this item.** | | ☒ \| **None** \| Click the tab key to add additional rows. | |
| **Time frame: past 36 months** | | | | |
| **2** | | Grants or contracts from any entity (if not indicated in item #1 above). | ☐ \| **None** National Institutes of Health \| Payments made to institution; Michael J Fox Foundation \| Payments made to institution | |
| **3** | | Royalties or licenses | ☐ \| **None** Lippincott Williams & Wilkins \| Medical textbooks; Cambridge University Press \| Medical textbooks; Springer \| Medical textbook | |
| **4** | | Consulting fees | ☐ \| **None** Mitsubishi Tanabe Pharma America (formerly, Neuroderm) \| Personal compensation as a consultant/scientific advisory board member; Herantis Pharma \| Personal compensation as a consultant/scientific advisory board member; Amneal \| Personal compensation as a consultant/scientific advisory board member; Abbvie \| Personal compensation as a consultant/scientific advisory board member; Acorda \| Personal compensation as a consultant/scientific advisory board member; Bial \| Personal compensation as a consultant/scientific advisory board member; Merz \| Personal compensation as a consultant/scientific advisory board member; NeuroDiagnostics, Inc (SYNAPS Dx) \| Personal compensation as a consultant/scientific advisory board member; Supernus (formerly, USWorldMeds) \| Personal compensation as a consultant/scientific advisory board member; Intrance Medical Systems, Inc. \| Personal compensation as a consultant/scientific advisory board member; Praxis Precision Medicines \| Personal compensation as a consultant/scientific advisory board member; Citrus Health \| Personal compensation as a consultant/scientific advisory board member | |
| **5** | | Payment or honoraria for lectures, presentations, speakers bureaus, manuscript writing or educational events | ☒ \| **None** | |
| **6** | | Payment for expert testimony | ☒ \| **None** | |
| **7** | | Support for attending meetings and/or travel | ☒ \| **None** | |
| **8** | | Patents planned, issued or pending | ☐ \| **None** Co-owner of patent “compositions and methods for treatment and/or prophylaxis of proteinopathies.” \| Cofounded REGAIN Therapeutics [relinquished any future payments], co-owner of the patent | |
| **9** | | Participation on a Data Safety Monitoring Board or Advisory Board | ☐ \| **None** Data and Safety Monitoring Board (DSMB), Open-Label Safety Study of Glial Cell Line-Derived Neurotrophic Factor Gene Transfer (AAV2-GDNF) in Parkinson’s Disease, GDNF-102 Phase 1b trial, AskBio, June 2020- \| Payment made to self as Chair of DSMB; Data and Safety Monitoring Board (DSMB), Glial cell line-derived neurotrophic factor (GDNF) Gene Therapy in for Multiple System Atrophy study, Protocol number MSA-101. AskBio, June 2021- \| Payment made to self as Chair of DSMB | |
| **10** | | Leadership or fiduciary role in other board, society, committee or advocacy group, paid or unpaid | ☒ \| **None** | |
| **11** | | Stock or stock options | ☒ \| **None** | |
| **12** | | Receipt of equipment, materials, drugs, medical writing, gifts or other services | ☒ \| **None** | |
| **13** | | Other financial or non-financial interests | ☒ \| **None** | |
|  | |  |  | |
| **Please place an “X” next to the following statement to indicate your agreement:** | | | | |
| ☒ | | I certify that I have answered every question and have not altered the wording of any of the questions on this form. | | |
